# Assessing worst case scenarios in movement demands derived from global positioning systems during international rugby union matches: Rolling averages versus fixed length epochs

**DOI:** 10.1371/journal.pone.0195197

**Published:** 2018-04-05

**Authors:** Daniel J. Cunningham, David A. Shearer, Neil Carter, Scott Drawer, Ben Pollard, Mark Bennett, Robin Eager, Christian J. Cook, John Farrell, Mark Russell, Liam P. Kilduff

**Affiliations:** 1 Applied Sport Technology Exercise and Medicine Research Centre (A-STEM), College of Engineering, Swansea University, Swansea, Wales; 2 School of Psychology and Therapeutic Studies, University of South Wales, Rhondda Cynon Taff, Wales; 3 Welsh Institute of Performance Science, College of Engineering, Swansea University, Swansea, Wales; 4 Department of Psychology, College of Human and Health Science, Swansea University, Swansea, Wales; 5 Sky Performance Hub, Team Sky, London, England; 6 Saracens RFC, North London, England; 7 The Rugby Football Union, Greater London, England; 8 University of Canberra Research Institute for Sport and Exercise, University of Canberra, Canberra, Australia; 9 Georgian Rugby Union, Tbilisi, Georgia; 10 School of Social and Health Sciences, Leeds Trinity University, Leeds, England; Nottingham Trent University, UNITED KINGDOM

## Abstract

The assessment of competitive movement demands in team sports has traditionally relied upon global positioning system (GPS) analyses presented as fixed-time epochs (e.g., 5–40 min). More recently, presenting game data as a rolling average has become prevalent due to concerns over a loss of sampling resolution associated with the windowing of data over fixed periods. Accordingly, this study compared rolling average (ROLL) and fixed-time (FIXED) epochs for quantifying the peak movement demands of international rugby union match-play as a function of playing position. Elite players from three different squads (*n* = 119) were monitored using 10 Hz GPS during 36 matches played in the 2014–2017 seasons. Players categorised broadly as forwards and backs, and then by positional sub-group (FR: front row, SR: second row, BR: back row, HB: half back, MF: midfield, B3: back three) were monitored during match-play for peak values of high-speed running (>5 m·s^-1^; HSR) and relative distance covered (m·min^-1^) over 60–300 s using two types of sample-epoch (ROLL, FIXED). Irrespective of the method used, as the epoch length increased, values for the intensity of running actions decreased (e.g., For the backs using the ROLL method, distance covered decreased from 177.4 ± 20.6 m·min^-1^ in the 60 s epoch to 107.5 ± 13.3 m·min^-1^ for the 300 s epoch). For the team as a whole, and irrespective of position, estimates of fixed effects indicated significant between-method differences across all time-points for both relative distance covered and HSR. Movement demands were underestimated consistently by FIXED versus ROLL with differences being most pronounced using 60 s epochs (95% CI HSR: -6.05 to -4.70 m·min^-1^, 95% CI distance: -18.45 to -16.43 m·min^-1^). For all HSR time epochs except one, all backs groups increased more (p < 0.01) from FIXED to ROLL than the forward groups. Linear mixed modelling of ROLL data highlighted that for HSR (except 60 s epoch), SR was the only group not significantly different to FR. For relative distance covered all other position groups were greater than the FR (p < 0.05). The FIXED method underestimated both relative distance (~11%) and HSR values (up to ~20%) compared to the ROLL method. These differences were exaggerated for the HSR variable in the backs position who covered the greatest HSR distance; highlighting important consideration for those implementing the FIXED method of analysis. The data provides coaches with a worst-case scenario reference on the running demands required for periods of 60–300 s in length. This information offers novel insight into game demands and can be used to inform the design of training games to increase specificity of preparation for the most demanding phases of matches.

## Introduction

Rugby union is a collision sport involving intermittent high intensity periods of play, where intense static exertions, collisions, and bouts of high speed running (HSR) are interspersed with random periods of lower intensity work and rest [1, 2]. To aid the specificity of team sport training, micro-sensor technology, such as Global Positioning Systems (GPS), is widely used to quantify the workloads of elite players during training and match-play [1, 3–9]. Such methods have highlighted that rugby union players cover 5–7 km per game [3, 10–12], with backs covering greater distances than forwards but sustaining less contact loads from scrums, rucks and mauls [10, 13]. Additionally, distinct differences also exist within players of the same general positional grouping (forwards/backs); for example, back row forwards have been reported to cover greater relative and HSR distances, produce more frequent high acceleration and deceleration events, and make more tackles than front row forwards [4, 10–12, 14]. Characterising a player’s movement patterns allows coaches a greater insight into the positional requirements of performance [4, 6] and consequently facilitates the planning, implementation and monitoring of training programmes that seek to achieve the desired physiological stress while minimising the risk of overtraining and injury [9, 15].

To date GPS studies have typically reported the average movement demands of each half or the full game [1–3, 5, 11, 12]. For example, a study of English Premiership rugby [1] highlighted a median total distance of 5850 m (64.6 m·min^-1^) and 6545 m (71.1 m·min^-1^) was covered by forwards and backs, respectively. Similar between-position differences exist for relative distance covered and HSR with values of 66.8 m·min^-1^ and 3.1 m·min^-1^ being covered by forwards versus 73.3 m·min^-1^ and 7.2 m·min^-1^ by backs, respectively [12]. Using 10 min fixed-time epochs, Jones and co-workers [4] reported transient changes throughout the duration of match-play with the greatest demands (relative distance covered) in the first 10 minutes of each half (i.e. 75.3 and 74.3 m·min^-1^ respectively); values which exceeded whole match averages (i.e., 66.2 m·min^-1^) and declined thereafter.

While such data has been advantageous for profiling the general demands of rugby match-play, it must be noted that the use of averages in such studies likely underestimates peak demands, and thus the worst case scenarios of the sport. To optimally prepare athletes for the demands of competition, it is vital that they are trained to cope with the most intense periods of the game and not just average demands [7, 15, 16]; especially for rugby which is more intermittent in nature [17, 18]. Although this might not be a direct performance issue, it could expose players to an increased risk of injury by exposing them to spikes in match-related workloads through omission of equivalent loads during training [7, 15]. The ability to devise training drills that concurrently develop physical qualities that equal or exceed the movement demands of match-play, whilst maintaining high skill levels and tactical emphasis, would represent an effective and stimulating training environment [19].

The use of smaller fixed-time epochs (e.g. 10 min) provides further insight into more demanding periods of the game [4]; however, this method has been shown to be inferior compared to a rolling average method for determining peak HSR distances during soccer matches [20]. Using five min fixed-time epochs (e.g., 0–5, 6–10 min etc.) and rolling averages of the same duration in elite soccer matches, Varley et al., [20] indicated a 20–25% underestimation of peak HSR distance and a 31% overestimation of HSR distance using fixed-time analyses. Similarly, in rugby sevens, a two min rolling average was used to assess the most demanding phases of the game in terms of relative distance covered and metabolic power [18]; with players covering 130 m·min^-1^ and producing 13 W·kg^-1^ during peak periods of match-play. Delaney and co-workers [17] used relative distance, average number of acceleration/decelerations and average metabolic power as their metrics to describe the peak running intensities (for rolling average epochs of 1–10 minutes) during international rugby union matches. Distances covered ranged from 184 m·min^-1^ (1 min epoch) for the half back group to 79 m·min^-1^ for the tight five group (10 min epoch); values which exceed those reported using average GPS values [1–5, 11, 12].

Therefore, the aim of the current study was to describe duration specific peak running intensities in two ways. Firstly, by comparing the rolling average method (ROLL) to fixed epoch method (FIXED) to calculate the greatest distance covered for each epoch length. Secondly to compare the ROLL method against the FIXED method when determining the greatest amount of HSR distance covered for each epoch length in international rugby union players. In addition, values for distance and HSR distance for each position and epoch length will be reported to give an insight into the ‘worst case scenario’ each position might be expected to face during competition.

## Methods

Elite professional players from three different international performance squads (*n* = 119) participated in the present study. Prior to providing written informed consent, participants were given information outlining the rationale, potential applications and procedures associated with the study. Study approval was granted by the Swansea University Ethics Committee. All players were considered healthy and injury-free at the time of the study and were in full-time training. Players were grouped as follows with the front row (FR), second row (SR) and back row (BR) positions making up the forwards (age: 24 ± 4 y; height: 188.5 ± 6.7 cm; body mass: 111.3 ± 9.3 kg) and half backs (HB), midfield/centres (MF) and back three (B3) positions making up the backs (age: 23 ± 4 y; height: 181.8 ± 6.3 cm; body mass: 90.0 ± 8.1 kg). A total of 708 GPS files from 36 international tournament games played from 2014–17 were collected. The high speed running (HSR) threshold was set at >5 m·s^-1^. Each player provided at least one GPS file with the largest number of files provided by any player being 13. A total of 148 GPS units were used during the study.

### Procedures

All matches took place between January 2014 and March 2017 where each player wore a GPS unit (Viper Pod, STATSport, Belfast, UK) in a bespoke pocket incorporated into their playing jersey on the upper thoracic spine between the scapulae to reduce movement artefacts [21]. The GPS units captured data at a sampling frequency of 10 Hz utilising the four best available satellites. Recent advancements in GPS technology have made 10 Hz units commercially available, which are more accurate than 1 & 5 Hz devices for quantifying movement patterns in team sports [22, 23]. Varley et al., [23] reported that a 10 Hz GPS unit was two to three times more accurate for instantaneous velocity during tasks completed at a range of velocities compared to a criterion measure, six times more reliable for measuring maximum instantaneous velocity, and had a coefficient of variation less than or similar to the calculated smallest worthwhile change [24] during all phases of acceleration and deceleration. More specifically this brand of GPS device has been used in team sports to assess movement demands during training and competitive matches [11, 12, 25–30]. In line with other brands of GPS these devices have been reported to display moderate validity over very short sprint distances (5 m) which increases to good over longer sprint distances (i.e., 20 m). On a practical level, this only amounted to an average of a 31 cm underestimation compared to the criterion method [31].

All participants were already familiarized with the devices as part of their day-to-day training and playing practices. Units were activated according to the manufacturer’s guidelines immediately prior to the pre-match warm-up (~30–60 min before kick-off), and to avoid inter-unit variation players wore the same GPS device for each match. Raw data files were then exported post-match and processed using a bespoke analysis program.

#### Rolling average and fixed length analysis

The analysis program generated measures averaged with two types of sample-epoch; rolling (ROLL) and fixed (FIXED) length epochs. Epoch length was specified by the user in seconds (60–300 s in increments of 60 s), and could be of arbitrary length. The actual length in samples was then calculated using the sampling rate, and allowed for missed samples. For instance, for a 60 s epoch-length with a sampling rate of 10 Hz, the epoch-length in samples was 600. Thus, for the rolling-epoch algorithm values were calculated using the current, and 599 preceding, samples. For the fixed-time method epochs were located at samples 1–600, 601–1200, 1201–1800, and so forth. From the speed (m·s^-1^) measure and sampling rate, distance (m) travelled since the previous sample was calculated. For both the ROLL and FIXED epoch analysis, for each input sample, a duplicate output sample was generated with added values for (1) the distance, (2) the distance covered above a speed of 5 m·s^-1^ (HSR), for the duration of the epoch.

#### Data analysis

The data consisted of repeated measurements of the same group of individuals across a number of matches (i.e., individuals nested with games). Therefore, to account for the interdependence of the data set and the risk of correlated error terms, linear mixed models were used to examine the differences in the dependent variables as a function of the method of measurement. In all models generated, random intercepts for both participant and game were prescribed to allow for the individuality of individuals, and the uniqueness of each game. Attempts to model random slopes for the same variables resulted in over specified models and was therefore abandoned.

To begin, separate multilevel models were run for each dependant variable at each time epoch (60–300 s) to examine differences in the FIXED and ROLL methods across the entire team (“method” entered as a fixed effect). Subsequently, position group (FR, SR, BR, HB, MF and B3) was entered as a fixed effect and assessment method (FIXED vs. ROLL) was entered as a covariate. This was done to simplify the interpretation of the interaction between position group and method. In all models, front row was used as a the baseline comparison, as it was deemed that this position group would most likely have the lowest values for both HSR and total distance across both methods, and would therefore allow for the examination of differences between position groups as a function of method.

## Results

For the whole team analysis, estimates of fixed effects indicated significant differences in values for each dependent measure across all time epochs as a function of analysis method (p < 0.001). Examination of beta estimates (Table 1), with the ROLL method as baseline, suggested the FIXED method always underestimated scores compared with the ROLL method (p < 0.001). For both HSR and total distance, the biggest (absolute) between-method differences occurred in the 60 s time epoch (Table 1), whereby beta estimates indicated that FIXED underestimated ROLL by 5.37 m·min^-1^ and 17.44 m·min^-1^ for HSR and distance covered, respectively. In relative terms, the difference between methods escalated as epoch length increased for HSR but not distance covered (Table 2).

**Table 1:** Estimates and confidence intervals for the differences in methods of measurement for the whole team (ROLL method as baseline).

				95% Confidence Interval	
Time epoch	Estimate	t	Sig.	Lower Bound	Upper Bound
HSR(60 s)	-5.37	-15.73	0.001	-6.05	-4.70
HSR(120 s)	-4.13	-18.02	0.001	-4.58	-3.68
HSR(180 s)	-3.95	-18.44	0.001	-4.37	-3.53
HSR(240 s)	-3.49	-18.79	0.001	-3.85	-3.12
HSR(300 s)	-3.06	-15.98	0.001	-3.44	-2.68
Distance(60 s)	-17.44	-33.86	0.001	-18.45	-16.43
Distance(120 s)	-12.87	-34.85	0.001	-13.60	-12.15
Distance(180 s)	-12.49	-38.50	0.001	-13.13	-11.85
Distance(240 s)	-11.19	-35.87	0.001	-11.80	-10.58
Distance(300 s)	-10.25	-36.34	0.001	-10.81	-9.70

**Table 2:** Relative distance and high speed running (HSR) covered by the forwards, backs and team and per cent differences between ROLL and FIXED methods.

HSR (m·min^-1^)	Team			Forwards			Backs		
Time epoch	ROLL Method	FIXED Method	Difference %	ROLL Method	FIXED Method	Difference %	ROLL Method	FIXED Method	Difference %
60 s	54.3 ± 25.1*^#^	49.0 ± 22.4^#^	-10.9	42.5 ± 20.6*	38.2 ± 17.5	-11.2	69.9 ± 21.8*	63.2 ± 20.2	-10.6
120 s	32.6 ± 17.6*^#^	28.5 ± 15.5^#^	-14.4	24.9 ± 15.0*	22.0 ± 13.3	-12.9	42.6 ± 15.7*	36.9 ± 14.0	-15.4
180 s	25.0 ± 15.6*^#^	21.1 ± 12.9^#^	-18.6	18.9 ± 14.0*	16.1 ± 11.3	-17.3	32.7 ± 14.0*	27.4 ± 12.0	-19.5
240 s	20.9 ± 13. 5*^#^	17.5 ± 11.1^#^	-19.8	15.5 ± 12.1*	13.2 ± 10.0	-17.6	27.6 ± 12.2*	22.8 ± 10.1	-21.3
300 s	17.9 ± 11.8*^#^	14.9 ± 9.1	-20.4	13.1 ± 10.2*	10.9 ± 7.3	-21.1	24.0 ± 10.8*	20.0 ± 8.5	-19.9
**Distance (m·min**^**-1**^**)**									
60 s	165.6 ± 22.3*#	148.1 ± 22.1#	-11.8	156.5 ± 19.0*	139.0 ± 38.2	-12.6	177.4 ± 20.6*	160.1 ± 21.1	-10.8
120 s	130.9 ± 17.8*#	117.9 ± 18.2#	-11.0	123.7 ± 15.4*	111.1 ± 22.0	-11.4	140.1 ± 16.3*	126.9 ± 16.7	-10.4
180 s	115.3 ± 16.5*#	102.8 ± 15.8#	-12.2	109.2 ± 14.6*	96.9 ± 16.1	-12.7	123.4 ± 15.4*	110.6 ± 15.0	-11.6
240 s	106.7 ± 15.0*#	95.5 ± 14.0#	-11.8	101.0 ± 12.9*	90.6 ± 13.2	-11.5	114.2 ± 14.4*	102.0 ± 13.4	-12.0
300 s	100.6 ± 14.0*	90.4 ± 13.9	-11.4	95.4 ± 12.2*	85.7 ± 10.9	-11.4	107.5 ± 13.3*	96.5 ± 13.6	-11.3

* = significant difference between ROLL and FIXED method.

^#^ = significant difference against 300 s epoch. Data presented as Mean ± S.D

For the linear mixed model with both method and positional group included, fixed effects indicated a significant main effect for positional group for all dependent measures (p < 0.001) indicating that positions differed in both HSR and total distance across all time epochs, irrespective of the method used. Fixed effects also indicated a significant (p < 0.01) interaction (position group * method) for all HSR measures (i.e., 60, 120, 180, 240, 300 s) and total distance (240 s). Estimates of fixed effects (Table 3) were further examined to explain the interactions, with FR as the baseline comparator. Beta estimates indicated that FR showed an increase from FIXED to ROLL methods in both HSR and total distance at all time epochs. As per the fixed effect, for all HSR time epochs (excluding one), B3, MF and HB increased significantly more (p < 0.01) from FIXED to ROLL than BR, SR and FR players. The one exclusion being HB for HSR (60 s) which came close to significance compared to FR (p = 0.06). For distance (240 s), only HB appeared to increase significantly more than FR players in terms of the increase from the FIXED to ROLL method.

**Table 3:** Estimates and confidence intervals for the interaction of position group and method.

					95% Confidence Interval	
GPS variable	Interaction	Estimate	t	Sig.	Lower Bound	Upper Bound
HSR(60 s)	Method * FR	3.71	5.52	0.00	2.39	5.03
	B3	3.43	3.25	0.00	1.36	5.51
	MF	4.19	3.40	0.00	1.77	6.61
	HB	2.01	1.87	0.06	-0.10	4.13
	BR	1.19	1.20	0.23	-0.77	3.15
	SR	0.77	0.65	0.52	-1.56	3.11
HSR(120 s)	Method * FR	2.54	5.70	0.00	1.66	3.41
	B3	2.99	4.35	0.00	1.64	4.35
	MF	3.05	3.77	0.00	1.46	4.63
	HB	3.51	4.99	0.00	2.13	4.89
	BR	0.74	1.13	0.26	-0.55	2.03
	SR	0.36	0.47	0.64	-1.16	1.89
HSR(180 s)	Method * FR	2.64	6.28	0.00	1.81	3.46
	B3	2.52	3.92	0.00	1.26	3.79
	MF	2.38	3.14	0.00	0.89	3.87
	HB	3.26	4.90	0.00	1.95	4.57
	BR	0.82	1.33	0.18	-0.39	2.04
	SR	-0.67	-0.90	0.37	-2.13	0.80
HSR(240 s)	Method * FR	2.37	6.55	0.00	1.66	3.09
	B3	2.43	4.39	0.00	1.34	3.52
	MF	2.33	3.55	0.00	1.04	3.61
	HB	2.80	4.91	0.00	1.68	3.92
	BR	0.16	0.30	0.76	-0.89	1.21
	SR	-0.42	-0.67	0.51	-1.68	0.83
HSR(300 s)	Method * FR	2.27	5.92	0.00	1.52	3.03
	B3	1.61	2.73	0.01	0.45	2.76
	MF	1.94	2.84	0.01	0.60	3.29
	HB	1.76	2.90	0.00	0.57	2.95
	BR	0.39	0.70	0.49	-0.71	1.50
	SR	-0.57	-0.85	0.40	-1.90	0.75
Distance(60 s)	Method * FR	16.91	16.59	0.00	14.91	18.92
	B3	-0.35	-0.21	0.83	-3.53	2.84
	MF	0.48	0.26	0.80	-3.19	4.15
	HB	1.25	0.77	0.44	-1.94	4.45
	BR	1.27	0.84	0.40	-1.71	4.24
	SR	0.69	0.37	0.71	-2.93	4.31
Distance(120 s)	Method * FR	12.90	17.36	0.00	11.44	14.36
	B3	0.03	0.03	0.98	-2.26	2.33
	MF	-0.86	-0.64	0.52	-3.51	1.79
	HB	1.35	1.15	0.25	-0.96	3.65
	BR	-1.29	-1.18	0.24	-3.45	0.86
	SR	0.96	0.73	0.46	-1.61	3.52
Distance(180 s)	Method * FR	11.76	18.21	0.00	10.49	13.02
	B3	0.66	0.64	0.52	-1.35	2.66
	MF	0.89	0.76	0.45	-1.42	3.20
	HB	1.68	1.63	0.10	-0.34	3.70
	BR	1.56	1.63	0.10	-0.32	3.43
	SR	-0.49	-0.42	0.67	-2.75	1.78
Distance(240 s)	Method * FR	10.64	17.11	0.00	9.42	11.86
	B3	0.32	0.33	0.74	-1.59	2.24
	MF	1.92	1.71	0.09	-0.29	4.13
	HB	2.85	2.89	0.00	0.91	4.79
	BR	-0.02	-0.02	0.98	-1.81	1.77
	SR	-1.28	-1.17	0.24	-3.43	0.87
Distance(300 s)	Method * FR	9.40	16.66	0.00	8.29	10.51
	B3	1.20	1.34	0.18	-0.55	2.94
	MF	1.72	1.67	0.10	-0.30	3.73
	HB	1.86	2.07	0.04	0.09	3.63
	BR	0.45	0.54	0.59	-1.19	2.09
	SR	0.77	0.76	0.45	-1.21	2.74

FR = front row; SR = second row; BR = back row; HB = half backs; MF = midfield, B3 = back three. HSR = high speed running (>5 m·s^-1^)

For the Linear mixed model with both method and unit (forwards v backs), fixed effects indicated a significant main effect for unit for all dependent measures (p < 0.001) indicating forwards and backs differed in both HSR and total distance across all time epochs, irrespective of the method used. Fixed effects also indicated a significant interaction (unit * method) for all HSR and distance measures indicating that scores differed within the forwards and backs as a function of the method used. Estimates of fixed effects (S1 Table) were further examined to explain the interactions, with the ROLL method used as a comparison baseline. Beta estimates indicated that backs and forwards all showed an increase from FIXED to ROLL methods in both HSR and total distances at all time epochs.

A linear mixed model was used to examine differences in HSR and total distance as a function of epoch length, and to examine the interaction between epoch length and method. Fixed effects indicated a significant main effect for epoch length (p < 0.001), and a significant interaction (p < 0.001) between epoch length and the method used for both HSR and distance. Follow on beta estimates (S2 Table) showed that values for both HSR and total distance increased significantly (p < 0.001) as epoch length decreased. Furthermore, results indicated that the FIXED method returned significantly lower values of both HSR and distance at all epoch lengths.

**Fig 1:**
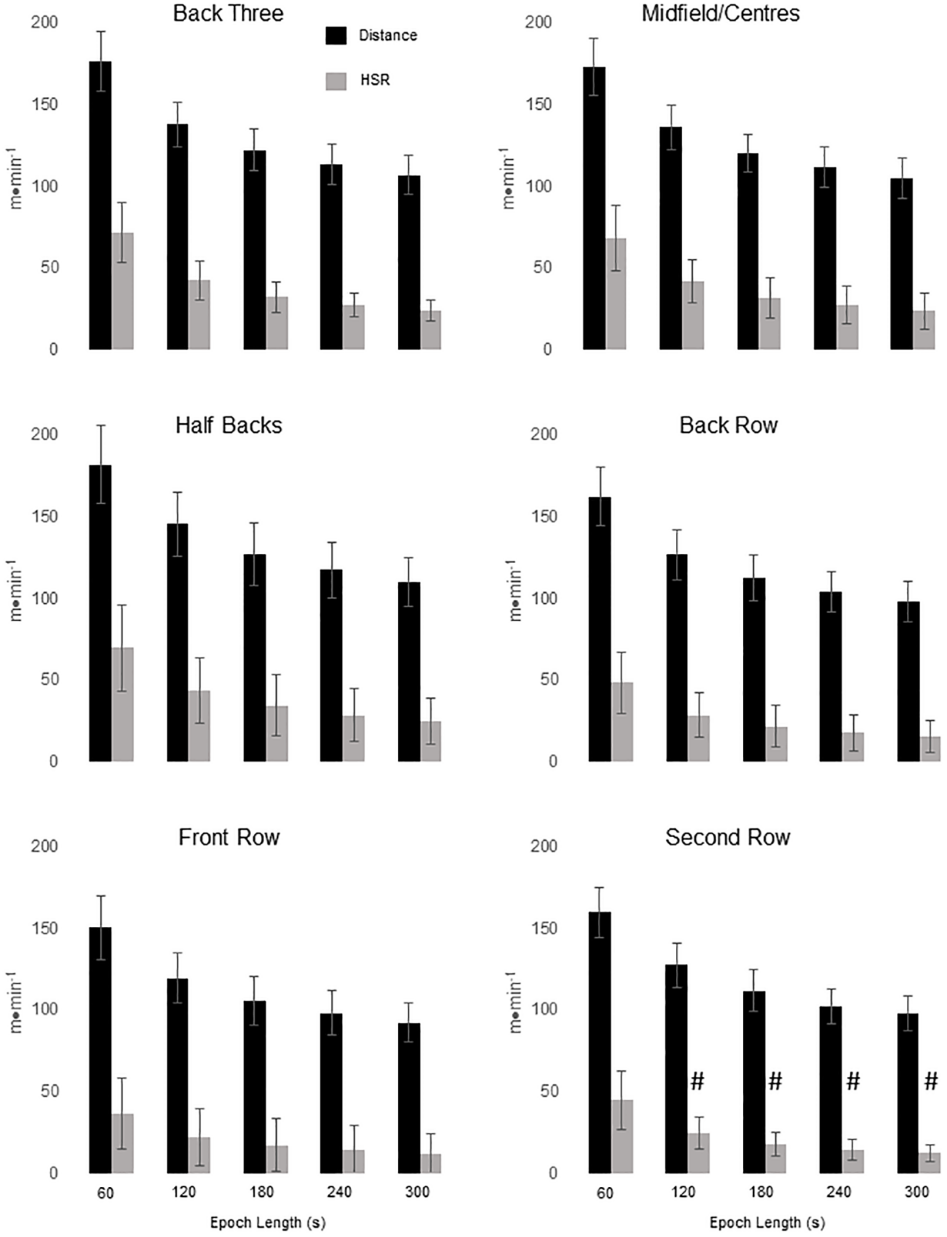
Rolling average method peak distance and high speed running (HSR) by position group for epoch lengths 60–300 s. ^#^ = significantly different to FR.

Given the consistent pattern of HSR and distance values being greater for the ROLL method, a final batch of linear mixed models were conducted to examine the differences between positional groups (Tables 4 and 5). Once again FR were used as the baseline to which all other positions were compared. For all but one HSR time epoch, HSR (60 s), beta estimates indicated that the only position not significantly different (p < 0.01) from the FR was SR (Fig 1). For HSR (60 s) all positions were significantly greater than FR. For all time epochs, beta estimates indicated that total distance for all positions was significantly greater (p < 0.05) than FR.

**Table 4:** Estimates and confidence intervals for HSR differences between positions using the ROLL method.

					95% Confidence Interval	
GPS variable		Estimate	*t*	*p*	Lower Bound	Upper Bound
HSR(60 s)	B3	34.70	13.35	0.00	29.60	39.81
	MF	31.57	10.57	0.00	25.70	37.43
	HB	32.65	12.34	0.00	27.45	37.84
	BR	11.42	4.66	0.00	6.61	16.23
	SR	8.03	2.74	0.01	2.28	13.79
	FR					
HSR(120 s)	B3	20.20	10.57	0.00	16.45	23.96
	MF	19.40	8.78	0.00	15.06	23.74
	HB	21.12	10.83	0.00	17.29	24.95
	BR	5.88	3.23	0.00	2.30	9.45
	SR	2.35	1.09	0.28	-1.88	6.59
	FR					
HSR(180 s)	B3	14.73	8.44	0.00	11.30	18.15
	MF	14.55	7.18	0.00	10.57	18.53
	HB	17.08	9.46	0.00	13.53	20.62
	BR	4.35	2.59	0.01	1.06	7.65
	SR	0.48	0.24	0.81	-3.50	4.46
	FR					
HSR(240 s)	B3	13.03	8.58	0.00	10.05	16.02
	MF	12.81	7.23	0.00	9.33	16.29
	HB	14.08	8.98	0.00	11.00	17.16
	BR	3.29	2.25	0.03	0.42	6.16
	SR	0.08	0.05	0.96	-3.37	3.53
	FR					
HSR(300 s)	B3	12.13	9.18	0.00	9.53	14.72
	MF	11.52	7.60	0.00	8.54	14.50
	HB	12.43	9.11	0.00	9.75	15.11
	BR	3.26	2.58	0.01	0.78	5.74
	SR	0.36	0.24	0.81	-2.62	3.34
	FR					

**Table 5:** Estimates and confidence intervals for relative distance differences between positions using the ROLL method.

					95% Confidence Interval	
GPS variable		Estimate	*t*	*p*	Lower Bound	Upper Bound
Distance(60 s)	B3	25.78	10.58	0.00	20.99	30.56
	MF	22.63	8.16	0.00	17.19	28.08
	HB	31.36	12.83	0.00	26.56	36.17
	BR	11.83	5.20	0.00	7.36	16.30
	SR	9.61	3.47	0.00	4.16	15.06
	FR					
Distance(120 s)	B3	18.20	9.25	0.00	14.34	22.07
	MF	16.73	7.44	0.00	12.32	21.15
	HB	25.84	13.05	0.00	21.95	29.73
	BR	7.25	3.92	0.00	3.62	10.88
	SR	8.13	3.69	0.00	3.80	12.46
	FR					
Distance(180 s)	B3	16.77	9.04	0.00	13.13	20.41
	MF	14.75	7.00	0.00	10.62	18.89
	HB	21.65	11.56	0.00	17.97	25.33
	BR	7.15	4.12	0.00	3.74	10.55
	SR	6.40	3.05	0.00	2.29	10.52
	FR					
Distance(240 s)	B3	14.99	8.81	0.00	11.65	18.33
	MF	13.49	6.95	0.00	9.68	17.30
	HB	19.07	11.08	0.00	15.69	22.46
	BR	5.66	3.56	0.00	2.54	8.79
	SR	3.87	2.02	0.04	0.11	7.62
	FR					
Distance(300 s)	B3	14.38	9.11	0.00	11.28	17.48
	MF	12.53	6.97	0.00	9.00	16.06
	HB	17.79	11.13	0.00	14.65	20.93
	BR	5.51	3.72	0.00	2.60	8.42
	SR	5.64	3.15	0.00	2.12	9.15
	FR					

## Discussion

The main aim of this study was to compare two different methods of assessing peak running intensities during international rugby union matches; namely, GPS analysis via rolling average and fixed-time epoch methods to ascertain the interchangeability of these methods such that the peak or ‘worst case scenario’ within-game running demands were identified. The primary finding was that the FIXED method underestimated both the maximum distance covered and HSR distance irrespective of epoch length (over 60–300 s) for the team and when split by positional group. Additionally, the increased HSR demands elicited from presenting data as ROLL compared to FIXED were greater for backs (i.e., HB, MF, B3) compared to FR; potentially, highlighting the insensitivity of FIXED for players that perform the highest levels of HSR during match-play [3, 4, 11, 12]. Backs covered greater total distances and HSR than the forwards regardless of the method used; a finding which agrees with previous literature [4, 11, 12, 17, 32]. Focusing on the ROLL method, the FR covered the least amount of distance for all epoch lengths compared to all other groups. Similarly, the FR covered the least amount of HSR for all epoch lengths (Fig 1) along with the SR group for epochs between 120–300 s. However, it is worth noting that this analysis only includes movement demands and does not consider the higher collision demands of the FR group and forwards in general [3, 13].

Data from elite soccer indicated that the use of fixed time epochs underestimated distance covered by up to ~25% compared to the rolling average method over a 5 min period [20]. In the current study using rugby players, the FIXED method underestimated by a more modest ~11–12% compared to the ROLL method and was consistent for all epoch durations. Both the ROLL and FIXED methods demonstrated that the peak intensities seen in competition were far greater than the average demands reported in previous literature irrespective of how this data was presented as either whole match, halves or even 10 min epochs [1–5, 11, 12, 33]. Jones and co-workers [4], utilised 10 min fixed epochs and reported similar peak values for distance and HSR covered for comparable epoch lengths (i.e., ~75–85 and ~7 m·min^-1^ vs 76.6 and 9.8 m·min^-1^ for relative distance and HSR respectively). However, from a practical/training prescription standpoint small changes in relative distance covered are unlikely to influence a coach’s prescription of training drills. Previous research in the area has utilised a cut off <10 m·min^-1^ between epoch lengths [17, 34, 35], reporting no additional practical benefit in examining epoch lengths >5 min in rugby codes. A similar trend was seen in the current study for the same epoch lengths (6–10 min), therefore analysis was stopped at 5 min (i.e., 300 s).

Lacome et al., [32] reported fixed peak five min periods for distance and HSR in international rugby union matches. Distances covered were greater than both the corresponding ROLL and FIXED epoch reported in our investigation. HSR distances reported for forwards (11.8 ± 7.8), and backs (19 ± 8 m·min^-1^) were similar to the FIXED method in the current study for both groups; however, our ROLL method produced higher values (13.1 ± 10.2 and 24.0 ± 10.8 m·min^-1^). We propose that the main reason for the discrepancies between the two studies could be due to differences in data collection technique as Lacome et al., [32] utilised an optical tracking (camera-based) system, whilst the current study used GPS devices. Regardless, values reported for time frames 5–10 mins, still under represent the most intense periods of play. For both ROLL and FIXED methods employed here, and in agreement with previous literature [17, 35, 36] as the epoch length decreases (i.e., below six min) substantial and significant increases in running intensities were seen with the FIXED method underestimating at every epoch length.

A rolling average analysis for distance, average acceleration and deceleration, and metabolic power was employed by Delaney and colleagues [17] in their study in elite rugby union players. Direct comparison with the current study for some position groups is difficult due to different groupings and metrics being chosen (e.g. tight five vs front row and second row, HSR vs metabolic power), however, comparable distance values were reported for common groups in both studies. In HB, distance covered in the 60 s epoch in the current study was 181.7 m·min^-1^ compared to 184 m·min^-1^ with both data showing very similar rates of decay as the moving average epoch increased up to 300 s (110.1 m·min^-1^ vs 108 m·min^-1^). This information is of practical significance to coaches as for drills >6 min the required relative intensity will remain reasonably constant but for every minute less a drill lasts a significant increase in intensity is required to match peak game intensity.

Recent work by Reardon and co-workers [13] investigating the movement and collision demands during the single longest ball in play bout, reported an average duration of 152–161 s. Comparison to the 180 s rolling average epoch in the current study for HB and BR yields similar distance covered results; being, 127 and 112 m·min^-1^ versus 123 and 112 m·min^-1^ in ~152–161 s for HB and BR, respectively. Notably, this similarity between studies is in stark contrast when HSR distances are examined as 34.3 and 21.6 m·min^-1^ covered in the current study for HB and BR (180 s) exceeds 8.1 and 6.0 m·min^-1^ reported previously. Differences in methods of HSR measurement (i.e., standardised >5 m·s^-1^ in the current study vs 60% of max velocity) may account for some of the differences reported. Likewise, gross distance covered maybe dictated by long ball-in-play periods, however, this is not the case for peak periods of HSR. Maximum velocity attained by each position group [13] is well below reported max in game velocities of rugby players [37], coupled with the relatively high number of collisions reported during these bouts may indicate that these are periods of repeated phase play. Whereas the peak HSR periods reported in the current study may occur during other phases of play (e.g. both attacking and defensive: transitions, line breaks & kick chases) where players will have the opportunity to obtain and maintain higher velocities (i.e. HSR).

This study was the first to describe the peak HSR demand of international rugby union competition using a rolling average method, and further contributes to the existing literature that has used peak relative distances [17]. The reporting of average demands during games drastically underestimates the most demanding periods of play and offers only a basic approach to coaches for designing training drills. The FIXED method underestimated the ROLL method for distance and HSR at all time points therefore its use in this type of analysis can be improved upon. The positional running profiles for epoch lengths 60–300 s provides information for coaches to help prescribe and monitor training loads, allowing exposure of players to more appropriate training stimuli representative of competitive matches. However, it must be noted that in the current study the collision element of the game was not assessed and must be considered when replicating match intensity training. Including the contact/collision elements may alter the identification of the most intense periods. Integrating skill and contact events into the peak running demands for both attacking and defensive periods could be useful when developing specific training drills to expose players to peak competition demands.

## Supporting information

S1 Table Estimates and confidence intervals for differences between forwards and backs as a function of method. (DOCX)

S2 Table Main effect and interaction beta estimates for window length as a function of method. (DOCX)
