# Supplementary material for: Assessing worst case scenarios in movement demands derived from global positioning systems during international rugby union matches: Rolling averages versus fixed length epochs
Source: PLoS One. 2018 Apr 5;13(4):e0195197. doi: 10.1371/journal.pone.0195197 (PMC5886488; doi:10.1371/journal.pone.0195197)
Supplement: S1 Table — (DOCX) [file pone.0195197.s001.docx]

**S1 Table. Estimates and confidence intervals for differences between forwards and backs as a function of method**

|  |  |  |  |  |  | 95% Confidence Interval | |
| --- | --- | --- | --- | --- | --- | --- | --- |
| GPS variable | Main effect | Interaction | Estimate | t | Sig. | Lower Bound | Upper Bound |
| HSR(60 s) | Back | FIXED | -6.71 | -3.94 | 0.00 | -10.06 | -3.37 |
|  |  | ROLL |  |  |  |  |  |
|  | Forwards | FIXED | -4.27 | -2.87 | 0.00 | -7.18 | -1.35 |
|  |  | ROLL |  |  |  |  |  |
| HSR(120 s) | Back | FIXED | -5.68 | -4.59 | 0.00 | -8.11 | -3.25 |
|  |  | ROLL |  |  |  |  |  |
|  | Forwards | FIXED | -2.85 | -2.61 | 0.01 | -4.99 | -0.70 |
|  |  | ROLL |  |  |  |  |  |
| HSR(180 s) | Back | FIXED | -5.35 | -4.86 | 0.00 | -7.50 | -3.19 |
|  |  | ROLL |  |  |  |  |  |
|  | Forwards | FIXED | -2.78 | -2.83 | 0.01 | -4.71 | -0.85 |
|  |  | ROLL |  |  |  |  |  |
| HSR(240 s) | Back | FIXED | -4.86 | -5.10 | 0.00 | -6.73 | -2.99 |
|  |  | ROLL |  |  |  |  |  |
|  | Forwards | FIXED | -2.32 | -2.72 | 0.01 | -3.99 | -0.65 |
|  |  | ROLL |  |  |  |  |  |
| HSR(300 s) | Back | FIXED | -3.97 | -4.99 | 0.00 | -5.54 | -2.41 |
|  |  | ROLL |  |  |  |  |  |
|  | Forwards | FIXED | -2.29 | -3.22 | 0.00 | -3.69 | -0.90 |
|  |  | ROLL |  |  |  |  |  |
| Distance(60 s) | Back | FIXED | -17.34 | -10.24 | 0.00 | -20.66 | -14.02 |
|  |  | ROLL |  |  |  |  |  |
|  | Forwards | FIXED | -17.54 | -11.88 | 0.00 | -20.44 | -14.64 |
|  |  | ROLL |  |  |  |  |  |
| Distance(120 s) | Back | FIXED | -13.18 | -9.44 | 0.00 | -15.92 | -10.44 |
|  |  | ROLL |  |  |  |  |  |
|  | Forwards | FIXED | -12.69 | -10.38 | 0.00 | -15.09 | -10.29 |
|  |  | ROLL |  |  |  |  |  |
| Distance(180 s) | Back | FIXED | -12.82 | -10.18 | 0.00 | -15.30 | -10.35 |
|  |  | ROLL |  |  |  |  |  |
|  | Forwards | FIXED | -12.29 | -11.20 | 0.00 | -14.45 | -10.14 |
|  |  | ROLL |  |  |  |  |  |
| Distance(240 s) | Back | FIXED | -12.28 | -10.76 | 0.00 | -14.51 | -10.04 |
|  |  | ROLL |  |  |  |  |  |
|  | Forwards | FIXED | -10.42 | -10.47 | 0.00 | -12.37 | -8.47 |
|  |  | ROLL |  |  |  |  |  |
| Distance (300 s) | Back | FIXED | -10.93 | -9.94 | 0.00 | -13.09 | -8.78 |
|  |  | ROLL |  |  |  |  |  |
|  | Forwards | FIXED | -9.75 | -10.15 | 0.00 | -11.64 | -7.87 |
|  |  | ROLL |  |  |  |  |  |
