# Supplementary material for: Assessing worst case scenarios in movement demands derived from global positioning systems during international rugby union matches: Rolling averages versus fixed length epochs
Source: PLoS One. 2018 Apr 5;13(4):e0195197. doi: 10.1371/journal.pone.0195197 (PMC5886488; doi:10.1371/journal.pone.0195197)
Supplement: S2 Table — (DOCX) [file pone.0195197.s002.docx]

**S2 Table. Main effect and interaction beta estimates for window length as a function of method**

|  |  |  |  |  |  | 95% Confidence Interval | |
| --- | --- | --- | --- | --- | --- | --- | --- |
| GPS variable | Main effect | interaction | Estimate | t | Sig. | Lower Bound | Upper Bound |
| HSR | 60 s |  | 36.60 | 89.78 | 0.00 | 35.80 | 37.40 |
|  | 120 s |  | 14.61 | 35.73 | 0.00 | 13.81 | 15.41 |
|  | 180 s |  | 6.95 | 16.94 | 0.00 | 6.14 | 7.75 |
|  | 240 s |  | 2.88 | 7.02 | 0.00 | 2.07 | 3.68 |
|  | 300 s |  |  |  |  |  |  |
|  |  | 60 s * FIXED | -5.37 | -13.31 | 0.00 | -6.16 | -4.58 |
|  |  | 60 s * ROLL |  |  |  |  |  |
|  |  | 120 s * FIXED | -4.13 | -10.16 | 0.00 | -4.93 | -3.33 |
|  |  | 120 s * ROLL |  |  |  |  |  |
|  |  | 180 s * FIXED | -3.96 | -9.66 | 0.00 | -4.76 | -3.15 |
|  |  | 180 s * ROLL |  |  |  |  |  |
|  |  | 240 s * FIXED | -3.48 | -8.50 | 0.00 | -4.29 | -2.68 |
|  |  | 240 s * ROLL |  |  |  |  |  |
|  |  | 300 s * FIXED | -3.07 | -7.47 | 0.00 | -3.88 | -2.27 |
|  |  | 300 s * ROLL |  |  |  |  |  |
|  |  |  |  |  |  |  |  |
| Distance | 60 s |  | 65.04 | 132.81 | 0.00 | 64.08 | 66.00 |
|  | 120 s |  | 30.15 | 61.42 | 0.00 | 29.19 | 31.12 |
|  | 180 s |  | 14.98 | 30.65 | 0.00 | 14.02 | 15.93 |
|  | 240 s |  | 6.08 | 12.41 | 0.00 | 5.12 | 7.03 |
|  | 300 s |  |  |  |  |  |  |
|  |  | 60 s * FIXED | -17.43 | -35.53 | 0.00 | -18.39 | -16.47 |
|  |  | 60 s * ROLL |  |  |  |  |  |
|  |  | 120 s * FIXED | -12.88 | -26.10 | 0.00 | -13.85 | -11.91 |
|  |  | 120 s * ROLL |  |  |  |  |  |
|  |  | 180 s * FIXED | -12.49 | -25.53 | 0.00 | -13.45 | -11.53 |
|  |  | 180 s * ROLL |  |  |  |  |  |
|  |  | 240 s * FIXED | -11.19 | -22.78 | 0.00 | -12.16 | -10.23 |
|  |  | 240 s * ROLL |  |  |  |  |  |
|  |  | 300 s * FIXED | -10.23 | -20.93 | 0.00 | -11.19 | -9.28 |
|  |  | 300 s * ROLL |  |  |  |  |  |
